# Effect of Age and Acute-Moderate Intensity Exercise on Biomarkers of Renal Health and Filtration

**DOI:** 10.3390/biology11040527

**Published:** 2022-03-30

**Authors:** Jeffrey S. Forsse, David Buckley, Ahmed Ismaeel, Kathleen A. Richardson, Autumn Oliver, Panagiotis Koutakis

**Affiliations:** 1 Department of Health Human Performance and Recreation, Baylor University, Waco, TX 76706, USA; katie_adair1@baylor.edu; 2 Kinesiology Department, Stephen F. Austin State University, Nacogdoches, TX 75962, USA; david.buckley@uta.edu (D.B.); aoliver@ulm.vcom.edu (A.O.); 3 Integrative Immunology Laboratory, University of Texas Arlington, Arlington, TX 76019, USA; 4 Clinical Muscle Biology Lab, Baylor University, Waco, TX 76706, USA; ahmed_ismaeel@baylor.edu; 5 Edward Via College of Osteopathic Medicine, University of Louisiana Monroe, Monroe, LA 71203, USA

**Keywords:** aerobic exercise, cardiometabolic diseases, renal health and filtration, urine epidermal growth factor, estimated glomerular filtration rate

## Abstract

**Simple Summary:**

In this research manuscript, the authors demonstrate that when assessing renal health and filtration after an acute bout of moderate-intensity aerobic exercise in healthy individuals of varying ages, only renal function is altered via contemporary biomarker cystatin C, indicating actual changes in renal filtration are not solely due to concentration changes seen in traditional markers of renal function. The clinical application of this study is understanding the value that maintaining good health through the middle-age years offers in the prevention of renal decline. Additionally, healthy individuals appear to have a limit on how high renal health and filtration can be obtained, and in the absence of cardiometabolic diseases, exercise minimally influences renal health and filtration.

**Abstract:**

Aerobic exercise elicits a multitude of physiological improvements in both healthy and diseased populations. However, acute changes in renal health and filtration with aerobic exercise remain difficult to quantify by traditional biomarkers to estimate glomerular filtration rate (eGFR). This study aimed to determine if an acute bout of moderate-intensity aerobic exercise transiently improves non-traditional biomarkers when compared to traditional biomarkers of renal health and filtration in individuals without cardiometabolic diseases. Thirty-nine participants (*n* = 18 men; *n* = 21 women; age 32.5 + 12.6 yr; height 171.1 + 11.4 cm; weight 78.7 + 15.6 kg; BMI 27.1 + 5.8) completed a single bout of moderate-intensity (50–60% HRR) aerobic exercise. Blood and urine samples were collected and compared before and post-exercise. Serum creatinine, urine epidermal growth factor (uEGF), uEGF/urine creatinine ratio (uEGFR), and cystatin C (CyC) were measured. In addition, eGFR-MDRD and the CKD-epidemiology equations were used to analyze renal clearance. Relative to pre-exercise measures: serum creatinine (*p* = 0.26), uEGF (*p* = 0.35), and uEGFR (*p* = 0.09) remained unchanged, whereas cystatin C (*p* = 0.00) significantly increased post-exercise. CyC eGFR was the only estimator of renal filtration to significantly change (*p* = 0.04). In conclusion, CyC is the only biomarker of renal health and filtration to significantly increase after aerobic exercise. Further investigation focused on sampling time and exercise-intensity is needed to solidify the current understanding of renal health and filtration.

## 1. Introduction

Renal health and filtration in healthy pre-chronic kidney disease (CKD) individuals is an area of growing interest and concern in the general population [1]. CKD affects an estimated 10 to 16% of the world population, with 1 in 3 adults being at risk for developing CKD [2]. The exact mechanisms and timeline of development in the beginning phase of CKD remain elusive, mainly due to (1) absence of signs and symptoms and (2) lack of early primary disease diagnosis (hypertension and diabetes) [2]. Traditionally, the biomarker serum creatinine (sCr) has been used to estimate glomerular filtration rate (eGFR) to assess renal filtration indirectly [3]. However, due to the volatile nature of sCr (e.g., affected by several factors, including muscle mass, diet, medications, mode and intensity of exercise, and hydration), there are limitations to using sCr to interpret renal filtration in healthy or undiagnosed individuals with kidney disease [4,5]. Additionally, sCr continues to be used to assess renal filtration due to its longstanding practice [6].

Recently, biomarkers cystatin C (CyC) and urine epidermal growth factor (uEGF) have been introduced as more reliable markers of kidney filtration and health when used in conjunction with sCr to quantify acute changes in renal health and filtration [7]. Over the last 20 years, CyC has been proposed as an added biomarker of renal filtration that can be used as a better estimator of glomerular filtration rate [8]. CyC is produced by all cell types, in all tissues, and is metabolized during glomerular filtration [9]. Therefore, CyC can be used as an indirect biomarker to assess acute fluctuations in renal filtration and function because CyC does not undergo renal tubular secretion. Instead, it is reabsorbed and catabolized by renal tubular cells [10]. However, the utilization of CyC is still under used in the clinical setting even with substantial supporting evidence [11]. Additionally, uEGF has been proposed to be a biomarker of renal health that could be used in conjunction with creatinine to more accurately distinguish between changes of renal health and acute changes in circulating sCr [12]. The kidneys produce uEGF, which initiates multiple intracellular pathways, stimulating renal cell growth, survival, and replication [13,14,15]. Unlike creatinine and cystatin C, uEGF is produced in the ascending portion of Henle’s loop and the distal convoluted tubules. Therefore, uEGF is potentially a more direct determinate of renal health [16,17,18]. Higher concentrations of uEGF are indicative of better renal health, with lower concentrations indicating poorer renal health [12].

One of the factors known to affect traditional markers of renal health and filtration is exercise [19]. In fact, aerobic exercise elicits a multitude of physiological improvements in both healthy and diseased populations [20,21]. Individuals who actively participate in various forms of aerobic exercise consistently exhibit improvements in cardiovascular and metabolic health regardless of health status [22,23]. However, the influence of aerobic exercise on renal health and filtration changes in healthy individuals free of early diagnosis of CKD and cardiometabolic disease is not fully characterized. Acute changes in renal health and filtration with aerobic exercise remain challenging to quantify in healthy populations due to undetermined mechanisms and inaccuracies of traditional biomarkers of renal health and filtration. Due to the limitations of the traditional biomarkers, acute changes with aerobic exercise are not fully understood. Multiple studies have shown acute exercise affects creatinine levels via exercise-induced muscle breakdown and dehydration [4,24]. Moreover, the impact of aerobic exercise on renal filtration has primarily been studied in competitive athletes and younger populations [25,26]. Importantly, age is also a factor in renal health and filtration [27]. However, it is often difficult to delineate the effects of aging from age-associated chronic diseases on renal filtration. For example, a pilot study from our lab showed that in the absence of cardiometabolic disease, age had no effect on markers of renal health and filtration [27]. Thus, it is important to determine the influence that aerobic exercise may have on renal health and filtration in normal, healthy, active populations across a wide age range, representative of the general population.

The purpose of this study was to determine the effect an acute bout of moderate-intensity aerobic exercise has on contemporary biomarkers CyC and uEGF when compared to traditional biomarker sCr. Our primary hypothesis is sCr will have a greater amount of volatility with aerobic exercise and not clearly represent actual changes in renal filtration and that CyC and uEGF will positively reflect actual changes in renal health and filtration with aerobic exercise. Our secondary hypothesis is age will not be an influencing factor in changes in renal health and filtration with an acute bout of aerobic exercise.

## 2. Materials and Methods

### 2.1. Participant Recruitment and Demographics

The participants recruited for this study were healthy, physically active, non-smokers, and between the ages of 20 and 60. A total of 39 individuals (males = 18 and females = 21) between the ages of 20 to 60 years of age participated in the study. Participant demographics are provided in Table 1. The American College of Sports Medicine and American Heart Association guidelines characterized a physically active definition: achieving the minimum exercise recommendations of 150 min per week of moderate-intensity exercise, or 75 min per week of high-intensity exercise [28,29]. Participants were currently not taking any medications except vitamins.

University Institutional Review Board (IRB) for research with human subjects (project # AY2018-1169) grant approval was obtained before the start of data collection. The research study and protocol adhere to the ethical guidelines outlined in the 1975 Declaration of Helsinki. All individuals who qualified for the study were provided both verbal and written material regarding the research study. The informed consent was authorized and returned by participants before admittance into the study.

Every participant completed a single health assessment to measure overall health status and quantify renal health and filtration. Participants arrived at the research lab after a minimum of a four- to six-hour fast but were told to consume water to maintain healthy hydration levels. A 12-oz. glass of water was offered to each participant immediately following the completion of the exercise protocol. Participants were instructed to abstain from exercise 24-h before the health assessment. Their baseline and exercise heart rate were recorded using a Polar H7 heart rate monitor (Polar, Bethpage, NY, USA). Blood pressure was obtained manually (American Diagnostic Corporation, Hauppauge, NY, USA) by experienced technicians. The same technicians obtained blood and urine samples under standardized conditions pre-and post-exercise. The samples were used to assess cardiometabolic health. All participants then completed the exercise intervention protocol.

### 2.2. Exercise Protocol

All participants completed the Gardner treadmill ramp protocol to achieve 55 to 60% of their heart rate reserve (HRR) [30]. The Gardner protocol was selected due to the current physical activity and fitness status of the general population. We used recommendations based on the American College of Sports Medicine recommendations for moderate-intensity exercise (40 to 60% VO_2_reserve) [29]. The exercise ramp protocol started at 2.0 mph and 0% grade and progressively increased by 2% grade every 2 min until 55 to 60% of HRR was achieved and maintained for 15 min. On average, individuals exercised for 20 min. A registered exercise physiologist completed all exercise tests.

### 2.3. Specimen Collection

Venous blood samples were obtained before the exercise protocol and 30 min after the exercise session was completed. The total amount of blood obtained from each participant was 15 mL and was obtained by venipuncture in the antecubital space. All blood samples were collected into 10 mL red-top (standard glass tube) and 4 mL purple top (KEDTA) sampling tubes. Hematocrit was obtained pre-and post-exercise to account for shifts in plasma volume due to sweat loss. Plasma tubes were allowed to clot for 30 min on ice. Both red- and purple-top tubes were then centrifuged at 3350 RPM for 15 min. Serum and plasma were retrieved from the red-top and purple-top tubes, individually, and were allocated into separate 2.0 mL storage tubes via transfer pipette and stored at −80 °C until all participants had completed the study.

Urine collection procedures consisted of research participants voiding their bladder fully into a sterile container. The specimen cup was returned to one of the study investigators. Urine collection occurred before and within 30 min after completing the exercise session. Upon collection, the sample was put on ice for 30 min then centrifuged for 5 min at 1000 RPM. The urine samples were separated into 2.0 mL plastic storage tubes and stored at −80 °C until analysis.

### 2.4. Biochemical Analysis

Biomarkers of renal filtration sCr and CyC concentrations were analyzed using the Piccolo Xpress blood chemistry analyzer Comprehensive Metabolica Panel (Abaxis, Inc., Union City, CA, USA) and a commercial ELISA kit (R&D Systems, Minneapolis, MN, USA and Arbor Assays, Ann Arbor, MI, USA), respectively. The intra-assay precision for the ELISA kit was determined as 3.1% coefficient of variation (CV). eGFR (mL/min/1.73 m^2^) was determined using equations validated by the National Kidney Foundation [31]. uEGF concentrations were determined using a commercial ELISA kit (R&D Systems, Minneapolis, MN, USA) with an intra-assay precision of 2.5% CV. Urine creatinine (uCr) concentrations were determined by a colorimetric detection kit (Enzo Life Sciences Inc., Farmingdale, NY, USA). Each serum, plasma, and urine sample were allowed to thaw to room temperature prior to analysis, and all samples, controls, and standards were assayed in duplicate. The optical density of Elisa kit wells was determined using an ELx808 absorbance microplate reader set to 450 nm (Biotek, Winooski, VT, USA). uEGF ratio (uEGFR) was log_2_ transformed to normalize the results [12]. Estimates of renal filtration were assessed using the Modification of Diet in Renal Disease (MDRD) and CKD-epidemiology equations using biomarkers SCr and CyC.

Renal Health Ratio = (uEGF/uCr) log_2_ (1)

### 2.5. Statistics

Responses to the exercise intervention were analyzed using a paired sample *t*-test. For additional statistical analyses, participants were divided into four groups (20s, 30s, 40s, and 50s). A 4 (age-group) × 2 (timepoints) analysis of variance (ANOVA) was performed to determine differences between groups and pre-and post-exercise means. A 2 (group 1: 20–39 and group 2: 40–60) × 2 (timepoints) ANOVA was also performed to account for smaller groups in the 30s and 50s age groups. Pearson’s coefficient of correlation (r) was calculated to assess the relationship between markers of renal health and filtration with age. Tukey’s post-hoc analysis was used to determine all possible pairwise comparisons since the sample sizes (groups) were unequal. Statistical significance was set at *p* ≤ 0.05. Descriptive statistics for participants are displayed as mean ± SD in Table 1. All other data are reported as mean ± SE. Data analyses were carried out using SAS software version 9.4 (SAS, Cary, NC, USA).

## 3. Results

Relative to pre-exercise measures: traditional and novel biomarkers of renal health and filtration sCr (*p* = 0.38), uEGF (*p* = 0.35), and uEGFR (*p* = 0.09) remained unchanged, whereas uCr (*p* = 0.045) and CyC (*p* = 0.0001) significantly increased post-exercise for the entire cohort. There was a difference in uEGF pre- to post-exercise, but the standard deviation was large, likely preventing uEGF from being significant. In conjunction, the MDRD (*p* = 0.08), CKD-EPI (*p* = 0.22), and sCr_CyC (*p* = 0.37) had no significant changes following the exercise intervention. However, average CyC eGFR significantly (*p* = 0.0004) decreased post-exercise (Table 2). To account for age and unequal number of individual group participants, individuals were divided up into group 1 (20–39 yrs.) and group 2 (40–59 yrs.) to determine any significant changes to exercise. A significant increase in CyC (*p* = 0.01) and a decrease in CyC_eGFR (*p* = 0.01) was observed for group 1 and a significant increase in CyC (*p* = 0.02) for group 2 (Table 2). No other changes were observed in groups 1 and 2. There was no statistical difference in hematocrit concentrations pre- to post-exercise. Therefore, fluid loss via changes in plasma volume shifts was not an influencing factor in biomarker outcomes.

**Figure 1:**
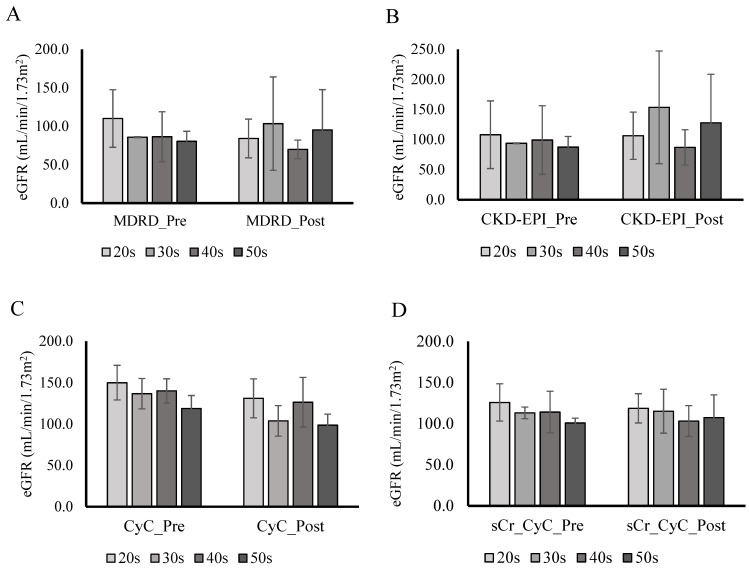
Age group comparison of eGFR (**A**–**D**). Abbreviations: Cystatin C (CyC), chronic kidney disease–epidemiology (CKD_EPI), estimated glomerular filtration rate (eGFR), Female (F), Male (M), Modification of Diet in Renal Disease (MDRD), serum creatinine (SCr), urine creatinine (uCR), and urine epidermal growth factor (uEGF). 20s—21 individuals (8 M/13 F), 30s—3 individuals (3 M), 40s—11 individuals (5 M/6 F), and 50s—4 individuals (2 M/2 F). All data are presented as mean + SD. There was no significant differences pre—to post-exercise.

The ANOVA revealed no significant differences in renal health and filtration between the four individual age groups pre-and post-exercise (Figure 1). The secondary ANOVA revealed there were no significant differences between group 1 and group 2 in all biomarkers of renal health and filtration. There were no statistical differences between gender. Pearson correlation revealed a significant negative correlation only between age and MDRD (r = −0.32, *p* = 0.04). Tukey’s multiple comparison post-hoc analysis was used to prevent inflating the type I error rate. Results of the post-hoc test revealed significant differences in CyC between the 20s and 50s age groups. All other post-hoc analyses showed no significant differences.

## 4. Discussion

This is the first study to show that an acute bout of moderate-intensity aerobic exercise in a wide age range of individuals free of cardiometabolic diseases elicited significant increases in renal filtration via biomarker CyC post-exercise, resulting in a decrease in eGFR. All other biomarkers of renal health and filtration concentrations were not significantly altered post-exercise. These data indicate that aerobic exercise may acutely decrease renal filtration, as measured by CyC. Therefore, CyC continues to be more directly linked to the precision of transient changes in renal filtration than traditional biomarker sCr.

Previous assessments of aerobic exercise’s influence on renal filtration in healthy populations have traditionally used sCr as the indirect biomarker to determine eGFR. In healthy populations, varying durations and aerobic exercise modes have led to conflicting influences on renal filtration [32,33]. Previously, two studies have shown that standard marathons led to elevated sCr levels post-race, which returned to baseline 24 h–5 days after [34,35]. In contrast, Poortmans et al. [5] demonstrated sCr remained stable during a triathlon. Interestingly, however, uCr was reduced after swimming, cycling, and running compared to baseline. Therefore, creatinine clearance may be insufficient to determine renal filtration changes following exercise interventions. Even with proper hydration, prolonged exercise with significant muscle damage increases creatinine concentrations acutely based on exercise intensity. In general, previous research studies observed the greatest change in serum creatinine concentrations and eGFR due to the intensity and length of the exercise session [25,32,33,34]. However, these changes in eGFR do not accurately reflect changes in renal filtration due to the high amount of protein degradation that influences serum creatinine concentrations. In our study, sCr, and eGFR calculated from sCr levels, were not significantly influenced by moderate-intensity aerobic exercise. This may be due to a minimal amount of muscle damage and fluid loss in the exercise protocol in our study, with exercise intensity not exceeding 60%.

Several studies have also used the CyC to determine eGFR following aerobic exercise bouts. CyC has been shown to increase after prolonged aerobic exercise in the hours post-exercise [25]. Likewise, when both CyC and sCr were measured in healthy male runners, both increased significantly after completing a marathon. Notably, however, sCr elevations were twice that of CyC. The increases in CyC may be transient, however, as CyC returned to baseline within 24 h post-marathon. Elevations in CyC typically occur immediately post-exercise and normally return to baseline within 24 h. On average, CyC increases 30% to 35% compared to baseline, while creatinine increases 50% to 60%, respectively. Previous studies involving CyC have studied young, healthy endurance athletes; however, our study population focused on young to older, generally active individuals. We observed that CyC significantly (*p* = 0.0001) increased following aerobic exercise, resulting in decreased eGFR (*p* = 0.0004), with no significant differences between age groups. On average, eGFR decreased 13.1% from baseline, however, post-exercise eGFR values were still within normal ranges (124.07 ± 31.82 mL/min/1.73 m^2^). Additionally, when using CyC solely to calculate eGFR instead of sCr, eGFR was, on average, 40% higher at baseline. These results support previous research that proposes utilizing CyC as a more reliable biomarker of renal filtration with aerobic exercise when compared to sCr due to its continued consistency as an indirect biomarker of renal filtration. Unlike previous studies that assessed changes in CyC with aerobic exercise, our study focused on the general population and the influence of age on renal outcomes. Thus, this indicates a potential ceiling effect in terms of what improvements in renal filtration can be obtained from participating in aerobic exercise.

Currently, only one study assessed changes in renal health via uEGF with aerobic exercise in healthy individuals. Konradsen et al. [36] studied the effect of a two-hour cross-country run on urine and blood epidermal growth factor levels in 25 healthy, trained individuals. Urine and blood were obtained to compare changes in renal health and filtration post-exercise. Urine samples were collected at baseline and within one-hour post-exercise. Blood samples were collected at baseline and immediately post-exercise. To adjust for dehydration, urine and blood samples were corrected for urine-specific gravity. uEGF increased 118% after exercise, while serum epidermal growth factor remained unchanged post-exercise compared to baseline [36]. When we applied the uEGF/uCr ratio to the Konradsen et al. [36] data, an acute increase of 4.2% in the uEGF/uCr ratio, indicating an improvement in this renal health marker, was observed. The significance of the uEGF/uCr ratio is the renal health/filtration comparison. The ratio appears to be lower in healthy populations than in known populations with renal dysfunction due to the high amounts of uCr filtered out of the blood and excreted into the urine [7,37]. The increases in uEGF production following aerobic exercise may be related to increased expression of the uEGF receptor [14]. The EGF receptor is centrally involved in stimulating cellular proliferation, migration, growth, and differentiation [38,39]. Various agonists such as GH, IL, and Angiotension II stimulate the EGF receptor, a G protein-coupled receptor, leading to the phosphorylation and activation of ADAM, which cleaves EGFR ligands, stimulating uEGF production [14,17]. Although our study outcome did not show any significant changes in the uEGF/uCr ratio, aerobic exercise intensity may be a crucial factor in stimulating uEGF activation. Forsse et al. [7] observed an increase in the uEGF/uCr ratio when implementing acute bouts of high-intensity interval exercise in individuals’ early stages of chronic kidney disease. An additional acute bout of aerobic exercise with continuous moderate-intensity exercise elicited no further significant uEGF/uCr ratio changes. Therefore, these two studies with diverse populations, coupled with our study results, support the notion that increases in the uEGF/uCr ratio may be stimulated by a sympathetic response, activated by different aerobic exercise modes with higher intensities.

Our secondary hypothesis of the study was based on age as an independent factor influencing changes in renal health and filtration with aerobic exercise. Our participant results were separated into one of four age groups (20s, 30s, 40s, and 50s) to assess differences in aerobic exercise influence on renal health and filtration based on age. There were no differences between age groups when combining novel and traditional biomarkers of renal health and filtration. Our results indicate a potential renal health and filtration threshold in normal, healthy kidneys that aerobic exercise does not transiently alter. These results suggest that in the absence of cardiometabolic diseases throughout young to middle-aged individuals, the glomeruli may have lower vascular and renal damage, indicating that age is more of an independent factor in renal decline [37]. In addition, it is essential to note that even the individuals in the older age groups in this study were physically active. Therefore, as recommended by the AHA and ACSM, regular aerobic exercise may play a role in maintaining renal health and filtration throughout the aging process. However, in the early stages of renal decline, aerobic exercise appears to improve renal health and filtration [7].

Limitations of the current study include (1) not assessing change in renal health and filtration for 24 h, (2) using indirect methods to assess renal filtration, (3) relying on participants to be honest and to abstain from exercise during the required time frame, (4) participants presenting accurate health records and information to be admitted into the study, (5) adhering to water consumption recommendations, and (6) unequal group sizes. Future studies should focus on standardizing and implementing recommendations focused on maintaining renal health and filtration with the aim of decreasing renal decline by preventing the development of cardiometabolic diseases known to impact kidney health negatively.

## 5. Conclusions

Our study demonstrates that an acute bout of moderate-intensity aerobic exercise has a minimal influence on novel and traditional biomarkers of renal health and filtration in young to middle-aged individuals free from CMD. There are three main conclusions to our study: (1) in healthy individuals, traditional markers of renal filtration and contemporary biomarker of renal health do not appear to be transiently affected by a short acute bout of moderate-intensity aerobic exercise; (2) biomarker CyC significantly increased with a short acute bout of moderate-intensity aerobic exercise; and (3) regardless of age, aerobic exercise has the same influence on renal health and filtration when cardiometabolic diseases are absent. Our study provides an in-depth focus for clinicians regarding the benefit of utilizing more novel biomarkers of renal health and filtration with traditional biomarkers to characterize acute changes more accurately. Additionally, healthy individuals appear to have a limit on how high renal health and filtration can be obtained and in the absence of cardiometabolic diseases, exercise minimally influences renal health and filtration.

## Figures and Tables

**Table 1:** Participant Demographics.

Variable	Mean	SD
**Entire Cohort**		
Age (yrs.)	33.4	12.5
Height (cm)	171.7	10.9
Weight (kg)	77.9	15.9
Body Mass Index (BMI)	26.5	5.5
Systolic Blood Pressure (SBP) (mmHg)	120.1	10.4
Diastolic Blood Pressure (DBP) (mmHg)	77.7	6.7
Heart Rate (HR) (beats per min)	70.0	12.2
Exercise HR (beats per min)	130.9	22.1
Estimated VO_2_ 50–60% (mL/kg/min^−1^)	28.7	4.2
Glucose (mg/dL)	95.4	7.3
Total Cholesterol (Chol) (mg/dL)	174.0	30.0
HDL (mg/dL)	55.0	18.0
LDL (mg/dL)	99.0	25.0
Albumin (g/dL)	4.0	0.3
Hematocrit (%)	45.0	4.0
**20s**		
Age (yrs.)	22.0	2.8
Height (cm)	172.2	9.4
Weight (kg)	76.6	15.2
Body Mass Index (BMI)		
Systolic Blood Pressure (SBP) (mmHg)	116.8	9.9
Diastolic Blood Pressure (DBP) (mmHg)	76.1	6.4
Heart Rate (HR) (beats per min)	72.4	11.0
Exercise HR (beats per min)	138.0	19.0
Estimated VO_2_ 50–60% (mL/kg/min^−1^)	29.5	4.1
Glucose (mg/dL)	94.3	4.9
Total Cholesterol (Chol) (mg/dL)	168.0	30.5
HDL (mg/dL)	56.9	18.1
LDL (mg/dL)	91.6	25.2
Albumin (g/dL)	4.0	0.3
Hematocrit (%)	43.5	3.9
**30s**		
Age (yrs.)	33.0	1.6
Height (cm)	177.3	5.3
Weight (kg)	84.2	18.3
Body Mass Index (BMI)		
Systolic Blood Pressure (SBP) (mmHg)	124.5	3.8
Diastolic Blood Pressure (DBP) (mmHg)	80.5	6.5
Heart Rate (HR) (beats per min)	69.8	11.8
Exercise HR (beats per min)	124.8	18.9
Estimated VO_2_ 50–60% (mL/kg/min^−1^)	27.7	3.9
Glucose (mg/dL)	98.5	5.7
Total Cholesterol (Chol) (mg/dL)	192.5	9.7
HDL (mg/dL)	50.8	19.4
LDL (mg/dL)	107.3	17.0
Albumin (g/dL)	4.1	0.4
Hematocrit (%)	48.8	0.8
**40s**		
Age (yrs.)	44.5	3.8
Height (cm)	167.1	16.0
Weight (kg)	78.9	13.2
Body Mass Index (BMI)		
Systolic Blood Pressure (SBP) (mmHg)	124.7	13.2
Diastolic Blood Pressure (DBP) (mmHg)	80.7	6.2
Heart Rate (HR) (beats per min)	69.5	16.9
Exercise HR (beats per min)	121.8	21.7
Estimated VO_2_ 50–60% (mL/kg/min^−1^)	27.5	3.4
Glucose (mg/dL)	97.3	9.4
Total Cholesterol (Chol) (mg/dL)	178.7	32.4
HDL (mg/dL)	53.5	23.3
LDL (mg/dL)	104.0	26.6
Albumin (g/dL)	4.0	0.2
Hematocrit (%)	45.3	3.4
**50s**		
Age (yrs.)	53.3	2.2
Height (cm)	167.1	6.1
Weight (kg)	84.0	18.6
Body Mass Index (BMI)		
Systolic Blood Pressure (SBP) (mmHg)	123.0	10.8
Diastolic Blood Pressure (DBP) (mmHg)	82.5	3.0
Heart Rate (HR) (beats per min)	66.5	6.2
Exercise HR (beats per min)	123.3	27.3
Estimated VO_2_ 50–60% (mL/kg/min^−1^)	28.3	5.6
Glucose (mg/dL)	93.3	9.9
Total Cholesterol (Chol) (mg/dL)	164.3	46.1
HDL (mg/dL)	52.6	20.0
LDL (mg/dL)	92.8	29.8
Albumin (g/dL)	3.7	0.9
Hematocrit (%)	44.3	2.5

Note: All values and presented as mean ± standard deviation (SD).

**Table 2:** Changes in concentrations and estimates of renal health and function biomarkers.

Variable	Baseline	Post-Exercise	Delta	t-Value	*p*-Value
**Entire Cohort**					
SCr (mg/dL)	0.92 ± 0.23	0.91 ± 0.23	1.1%	−1.15	0.259
uCR (mg/dL)	145.5 ± 104.5	178.1 ± 138.7	22.4%	2.07	0.046 *
CyC (ng/mL)	611.82 ± 80.15	714.5 ± 167.12	16.8%	4.24	0.0001 *
uEGF (ng/mL)	6574.16 ± 1519.64	6780.0 ± 1467.1	3.1%	0.94	0.353
uEGF/uCR (ng/mL)/(mg/dL)	1.56 ± 0.81	1.94 ± 1.33	24.4%	1.71	0.096
eGFR (MDRD)	98.46 ± 35.12	83.41 ± 32.60	15.3%	−1.75	0.089
eGFR (CKD-EPI)	102.34 ± 45.78	107.90 ± 53.35	5.5%	1.23	0.229
eGFR (CyC)	142.80 ± 21.87	124.07 ± 31.82	13.1%	−3.94	0.0004 *
eGFR (CyC and SCr)	118.97 ± 23.05	113.47 ± 25.58	4.6%	−0.96	0.346
**Group 1 (20–39)**					
SCr (mg/dL)	0.96 ± 0.24	0.92 ± 0.25	3.4%	1.107	0.280
uCR (mg/dL)	150.68 ± 105.30	195.86 ± 144.67	29.9%	−1.97	0.061
CyC (ng/mL)	614.83 ± 83.13	730.92 ± 185.50	18.9%	−3.70	0.001 *
uEGF (ng/mL)	6717.43 ± 1540.43	6934.24 ± 1436.27	3.2%	−0.782	0.442
uEGF/uCR (ng/mL)/(mg/dL)	1.54 ± 0.84	1.83 ± 1.01	18.83%	−1.75	0.094
eGFR (MDRD)	105.67 ± 37.02	87.47 ± 32.18	17.2%	1.81	0.084
eGFR (CKD-EPI)	103.1 ± 42.12	114.57 ± 52.68	11.1%	−1.05	0.303
eGFR (CyC)	147.74 ± 22.03	126.57 ± 32.55	14.3%	4.12	0.001 *
eGFR (CyC and SCr)	122.71 ± 21.97	118.13 ± 25.59	3.7%	1.11	0.280
**Group 2 (40–59)**					
SCr (mg/dL)	0.93 ± 0.18	0.89 ± 0.19	4.3%	0.616	0.551
uCR (mg/dL)	137.69 ± 101.14	153.92 ± 123.48	11.8%	−1.24	0.235
CyC (ng/mL)	616.32 ± 77.16	704.43 ± 129.78	14.3%	−2.63	0.020 *
uEGF (ng/mL)	6255.81 ± 1420.11	6458.86 ± 1441.45	3.2%	−0.64	0.535
uEGF/uCR (ng/mL)/(mg/dL)	1.59 ± 0.74	1.64 ± 0.95	3.1%	−0.43	0.672
eGFR (MDRD)	78.22 ± 14.24	75.79 ± 31.52	3.1%	0.278	0.786
eGFR (CKD-EPI)	84.64 ± 20.95	97.92 ± 52.32	15.7%	−0.919	0.378
eGFR (CyC)	132.69 ± 18.55	116.90 ± 29.77	11.9%	1.84	0.088
eGFR (CyC and SCr)	105.91 ± 12.56	104.36 ± 21.71	1.5%	0.205	0.841

Abbreviations: Cystatin C (CyC), chronic kidney disease—epidemiology (CKD_EPI), estimated glomerular filtration rate (eGFR), modification of diet in renal disease (MDRD), serum creatinine (SCr), urine creatinine (uCR), and urine epidermal growth factor (uEGF). All eGFR equations are presented as mL/min/1.73 m^2^ units. All data are presented as mean + standard error (SE). * Indicates significant *p* < 0.05 differences pre—to post-exercise.

## Data Availability

Not applicable.
